# A Computerized Pharmacy Decision Support System (PDSS) for Headache Management: Observational Pilot Study

**DOI:** 10.2196/35880

**Published:** 2022-11-25

**Authors:** Serge Perrot, Anne-Priscille Trouvin, Beatrice Clairaz-Mahiou, Grégory Tempremant, François Martial, Diane Brément, Asmaa Cherkaoui

**Affiliations:** 1 Centre de la douleur Hôpital Cochin Paris France; 2 Société Francophone des Sciences Pharmaceutiques Officinales Paris France; 3 Regional Unions of Health Professionals, Pharmaciens Hauts-de-France Lille France; 4 Regional Unions of Health Professionals, Pharmaciens Nouvelle Aquitaine Bordeaux France; 5 Global Digital Innovation Sanofi Gentilly France; 6 Public Affairs Sanofi Consumer Healthcare Gentilly France

**Keywords:** headache, pharmacy, counselling, over-the-counter (OTC) medication, self-medication, decision support system, patient perception

## Abstract

**Background:**

Headaches are common and often lead patients to seek advice from a pharmacist and consequently self-medicate for relief. Computerized pharmacy decision support systems (PDSSs) may be a valuable resource for health care professionals, particularly for community pharmacists when counseling patients with headache, to guide treatment with over-the-counter medications and recognize patients who require urgent or specialist care.

**Objective:**

This observational pilot study aimed to evaluate a newly developed PDSS web app for the management of patients seeking advice from a pharmacy for headache. This study examined the use of the PDSS web app and if it had an impact on patient or pharmacy personnel counseling, pharmacy personnel perception, and patient perception.

**Methods:**

The PDSS web app was developed according to Francophone des Sciences Pharmaceutiques Officinales (SFSPO) recommendations for headache management, and was made available to pharmacies in 2 regions of France: Hauts de France and New Aquitaine. Pharmacy personnel received 2 hours of training before using the PDSS web app. All people who visited the pharmacies for headache between June 29, 2020, and December 31, 2020, were offered an interview based on the PDSS web app and given information about the next steps in the management of headaches and advice on the proper use of their medication. Patients and pharmacy personnel reported satisfaction with the PDSS web app following consultations or during a follow-up period (January 18 to 25, 2021).

**Results:**

Of the 44 pharmacies that received the PDSS web app, 38 pharmacies representing 179 pharmacy personnel used the PDSS web app, and 435 people visited these pharmacies for headache during the study period. Of these, 70.0% (305/435) asked for immediate over-the-counter analgesics for themselves and consulted with pharmacy personnel with the use of the PDSS web app. The majority of these patients were given advice and analgesics for self-medication (346/435, 79.5%); however, 17.0% (74/435) were given analgesics and referred to urgent medical services, and 3.5% (15/435) were given analgesics and referred to their general practitioner. All pharmacy personnel (n=45) were satisfied or very satisfied with the use of the PDSS web app, and a majority thought it improved the quality of their care (41/44, 93.2%). Most pharmacy personnel felt that the PDSS web app modified their approach to management of headache (29/45, 64.4%). Most patients were very satisfied with the PDSS web app during their consultation (96/119, 80.7%), and all felt mostly or completely reassured.

**Conclusions:**

Use of the PDSS web app for the management of patients with headache improved the perceived quality of care for pharmacy personnel and patients. The PDSS web app was well accepted and effectively identified patients who required specialist medical management. Further studies should identify additional “red flags” for more effective screening and management of patients via the PDSS web app. Larger studies can measure the impact of the PDSS web app on the lives of patients and how safe or appropriate pharmacy personnel recommendations are.

## Introduction

Self-medication with nonprescription or over-the-counter (OTC) medications represents a growing challenge to community pharmacies worldwide, especially in the context of pain management [1]. This is due to the risk of harm to the patient, which could occur following an incorrect self-diagnosis, inappropriate use of medication, side effects of the medications used, or interactions with concurrent medications [2]. Overuse of symptomatic medication can even lead to chronification of the condition, giving rise to chronic medication-overuse headaches from previously acute conditions [3]. Community pharmacies represent the ideal location for prevention of these risks [1].

The Global Burden of Disease study has ranked headache disorders as the second leading cause of years lived with disability worldwide [4], and pharmacists may be the first or only health care professionals to advise patients about self-medication [5]. Previous studies using simulated patient methodology have demonstrated the role of community pharmacists in self-medication. The studies suggest that pharmacists have a clear overview of medications taken by the patient, with pharmacists being easily accessible and ideally placed to identify inappropriate self-medication [6]. In the context of headache and migraine management, pharmacists may be considered crucial in their management, but only if they have a clear understanding of patient symptoms and are able to recommend an appropriate analgesic treatment or refer the patient for further care, either with the patients’ general practitioner (GP) or a specialist [7].

Pharmacy decision support systems (PDSSs) are often paper-based and have value in this form; however, a computerized PDSS can be a valuable tool to aid patient-pharmacist counseling and guide the information gathered by the pharmacist. For example, in a study by Bertsche et al [8], the authors used a PDSS for the counseling of patients with allergic rhinitis and conjunctivitis requiring OTC medications in a method similar to that used in this study. The authors found that the PDSS efficiently guided the pharmacist through counseling and, without use of the PDSS, the questions most commonly missed were those that would have informed the need for a referral to other health care services [8]. Similar tools are being developed in other therapy areas; for example, a cross-platform web app in an initial pilot has been shown to enhance pharmacist care for lower back pain [9], and authors of migraine studies have suggested that digital tools, such as trackers, may aid medical communication and optimize management [10]. Advantages of a computerized PDSS in the form of a web app include that this is easily accessed by pharmacies worldwide, enables consistency across many pharmacies, and will create a data repository that can be used for further analysis. These digital health tools aid the pharmacy in patient management and have the potential to reach the many patients visiting pharmacies each day and support the changing role of community pharmacists. Community pharmacy practice has changed in recent years from being “product-oriented” to “service-oriented” across most European countries in light of changes in national and European legislation as well as pharmacist or pharmacy organizations; the focus on the patient in pharmaceutical care has increased, in part due to the introduction of pharmacy principles for appropriate medication use and preventative care [11]. Considering the influence of OTC medications on societal health, more pharmacists and pharmacy personnel are being provided opportunities to expand their contribution to facilitate patients’ self-care [12]. This relieves the pressure on doctors who are receptive to pharmacists supporting patients with OTC diagnoses, and likewise, pharmacists are receptive to engaging more in a medical expert role [13]. The scope of clinical pharmacy practice across France alone is rapidly expanding in order to accommodate more public health issues and meet the challenges raised in areas affected by medical desertification—the inadequate access to health care for the population.

We are not aware of any specific algorithm-based computerized PDSSs for the management of headache disorders in the context of a primary care setting such as a community pharmacy. Therefore, this observational pilot study evaluates the use of a newly developed computerized PDSS platform (web app), based on previously published algorithms for the management of headache by pharmacists [14], to guide patient-pharmacy personnel counseling and referrals to appropriate medical services, dispensation of appropriate analgesic treatments, and identification of patients who require specialist care. The study aims to examine whether patient pathways, pharmacist satisfaction, and patient satisfaction are impacted by use of the PDSS web app.

## Methods

### Study Design

This was an observational pilot study designed to evaluate the impact of a PDSS web app on the management of headache by pharmacy personnel and the satisfaction of pharmacy personnel and patients. The PDSS web app was used for consultations between pharmacy personnel and patients in 2 regions of France: New Aquitaine (from June 29, 2020, to December 31, 2020) and Hauts de France (from September 1, 2020, to December 31, 2020). Pharmacists and patients reported their satisfaction with the PDSS web app during these times and during a follow-up period from January 18 to 25, 2021. The start date of the study was delayed in Hauts de France due to the effects of the COVID-19 pandemic.

### Ethical Considerations

This study was conducted in compliance with the provisions of Law no. 78-17 of January 6, 1978, relating to the processing of data, files, and freedoms, and those of the EU General Data Protection Regulation (GDPR) 2016/679 of May 25, 2018; security measures were put in place to protect personal data. Due to the observational nature of the study and according to the criteria defined by article L1123-7 of the French Public Health Code, it was not necessary to seek approval from an ethics board or the Comité de Protection des Personnes (committee for the protection of individuals).

### Patient Population

There was no selection of patients; demographic data of all patients visiting a selected pharmacy for headache were collected. Patients requiring OTC analgesics for immediate pain relief were asked for consent to participate by pharmacy personnel before proceeding with their consultation using the PDSS web app. Patients were systematically informed of the approach to manage their headache and could refuse to participate.

### Development of the PDSS Web App

The PDSS web app was developed in partnership with Sanofi, following a validation process for developing and testing apps based on the Good Automated Manufacturing Practice (GAMP 5) guidelines and the US Food and Drug Administration (Code of Federal Regulations 21 Part 11) and the European Union (EU Annex 11) regulations. An independent computerized system quality expert closely followed the validation process to ensure specification and testing activities were adequately covered and in accordance with the involved functional risks. The PDSS web app management algorithms are based on national French guidelines and have been previously published by the Société Francophone des Sciences Pharmaceutiques Officinales (SFSPO; French Scientific Pharmacist Society; Multimedia Appendices 1 and 2) [15]. Briefly, a multidisciplinary group including pharmacists, pharmacy students, patient associations, doctors, and members of the French Scientific Society for Pain (SFETD) contributed to the development of the SFSPO recommendations using the management algorithms [14] and the principles of design thinking, a working method that focuses on collective intelligence, human-centered research, and deep understanding for end user needs [16].

Development of the PDSS and digitalization to a computer web app was performed by a digital health provider (Observia). Development and testing of the PDSS web app followed good practice guidelines related to computerized systems; a plan test and independent computerized system quality expert test were carried out to identify scenarios and perform tests of nondeviation. Pharmacies tested the PDSS web app, and the final version was adopted after 3 steps of validation and quality assessment. A digital training program was developed for this study for the pharmacy personnel by *Le Monde Pharmaceutique* led by professors from the University of Bordeaux. The program was delivered over 2 hours of online training to include three topics: (1) the science of headache; (2) understanding the “red flags,” recommendations, and the questions to ask the patients; and 3) how to use the PDSS web app.

### Evaluation of the PDSS Web App

The study protocol and PDSS were validated by SFSPO and the regional presidents of the Regional Unions of Health Professionals (URPS); both are authorized by law to propose and conduct experiments and to implement health procedures at a national level if the results are conclusive. Pharmacists were solicited by the URPS presidents for the region, were informed, and volunteered to participate. URPS validated the pharmacies that volunteered. Personnel (including pharmacists, team members, and pharmacy assistants) at all selected pharmacies were trained to use the PDSS web app before using it for patient consultations to inform patients about the next steps in headache management (including hygiene and dietary practices, treatments, and their interactions and contraindications). A training certificate was sent to URPS following completion of the training, and only after receiving URPS approval was the pharmacy able to register on the PDSS web app. Participating pharmacies received financial compensation for the additional time spent during consultations via the regional health agency, representing the Ministry of Health (Agences Régionales de Santé [ARS]) in Hauts de France or via the URPS in New Aquitaine. Patients did not receive any incentives for participation.

Patient and pharmacy personnel perception of the PDSS web app were assessed via an online or paper satisfaction assessment questionnaire, which was developed by the SFSPO and validated by the URPS. Pharmacists were asked to complete the questionnaire after every 5 consultations, and all active pharmacies were contacted to answer the questionnaire over the phone during the follow-up period. Pharmacies were either asked to answer 9 questions (if they had previously completed any questionnaire) or 12 questions (if they had not previously answered any questionnaire). Patients were offered the option to complete the questionnaire after every consultation either on paper, or via QR code or website link.

### Study End Points

End points were reported by the patients and the pharmacy personnel. End points from the PDSS web app to guide the management of headache included concomitant medications, pain history, pain intensity, pain location, comorbidities, and drug contraindications. Pain intensity was reported by the patient either using a visual analog scale (VAS; 1 representing “no pain” to 10 representing “severe pain”) or a simple verbal scale (SVS; 0 representing “absent,” 1 representing “mild,” 2 representing “moderate,” 3 representing “intense,” and 4 representing “extremely intense”). All patients and pharmacy personnel who used the PDSS web app were asked to complete a perception and satisfaction questionnaire on the usefulness of the PDSS.

## Results

### Development of the PDSS Web App

**Figure 1:**
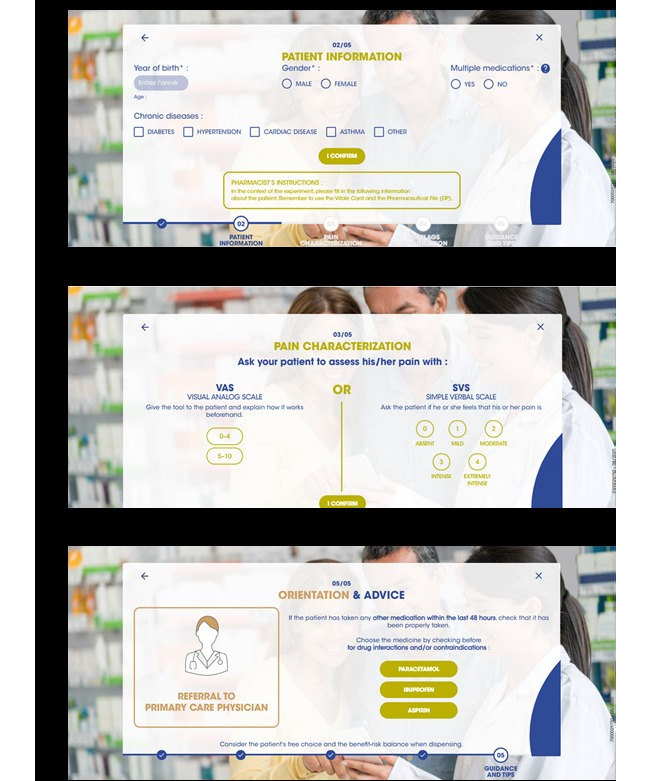
Screenshots of the pharmacy decision support system web app showing examples of information gathered during pharmacist-patient consultations: (a) patient characteristics, (b) patient-reported severity of pain, and (c) recommendations for referral to other medical services.

The PDSS web app was developed with up to 15 questions to be asked during patient-pharmacy personnel consultations and an estimated completion time of 4 minutes (Figure 1). Of the 435 consultations, the majority (n=346, 79.5%), were shorter than 4 minutes.

### Patient Demographic and Pain Characteristics

**Figure 2:**
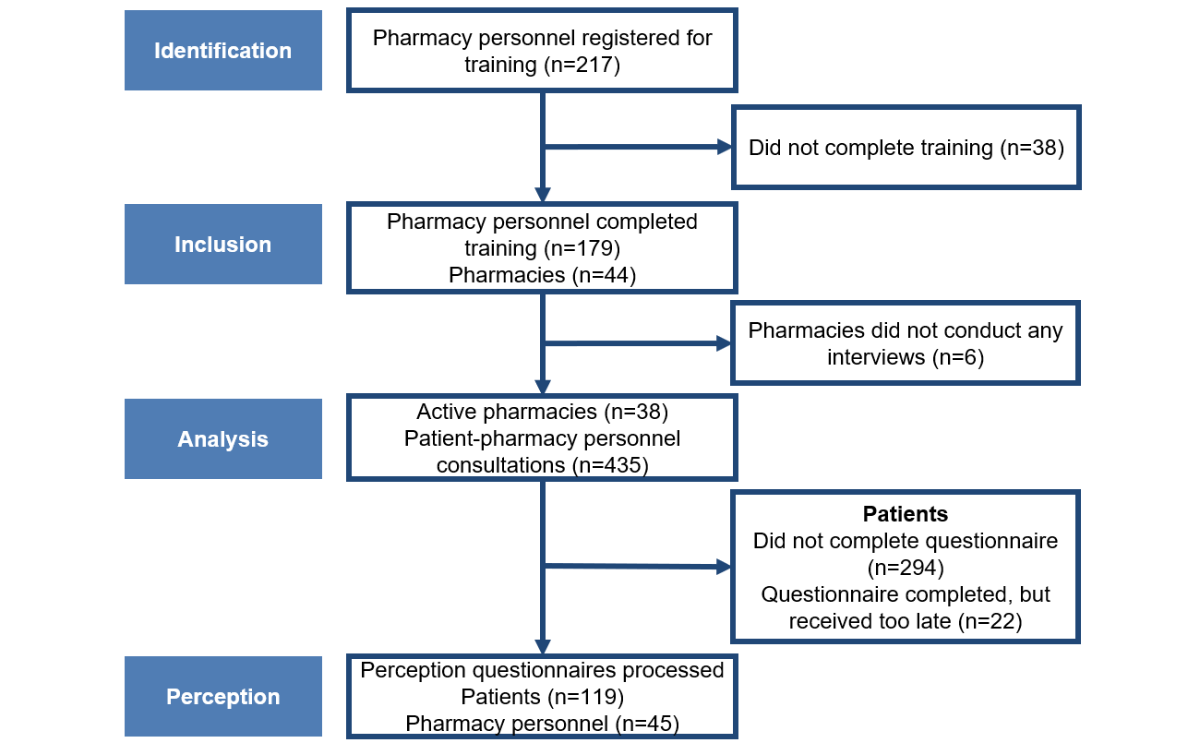
STROBE (Strengthening the Reporting of Observational studies in Epidemiology) flowchart.

A total of 69 pharmacies (Hauts de France n=46; New Aquitaine n=23) registered for the training, representing 217 pharmacy personnel (Figure 2). Of these, 44 pharmacies (Hauts de France n=32; New Aquitaine n=12), representing 179 pharmacy personnel, completed the training and were consequently selected to use the PDSS web app. Pharmacy personnel were generally satisfied (n=112, 62.5%) or very satisfied (n=56, 31.3%) with the training for the PDSS web app. At the end of the study period, 38 pharmacies (Hauts de France n=29; New Aquitaine n=9) had received visits from 435 patients or people acting on behalf of patients asking for relief from headache. Of the 435 people who visited the pharmacy, the majority were female (308, 70.1%) and under 50 years of age (mean age 43 years).

Out of the 435 people who visited the pharmacy for headache, 305 required immediate pain relief for their own self-medication, and 130 were acting on behalf of the patient. People visiting the pharmacy on the behalf of the patient were not asked questions with the PDSS web app beyond the first question; instead, they were given an information sheet for the patient and asked some questions to guide the recommendation (Multimedia Appendix 1): either an OTC analgesic to give to the patient or a recommendation for the patient to see their GP. Of the 435 people who visited the pharmacy for headache, 17.9% (78/435) were already prescribed >2 medications for other health issues (such as hypertension, diabetes, cardiovascular diseases, and asthma). Of the 305 patients in need of immediate pain relief for their own headache, 35.7% (109/305) of patients were already regularly taking analgesic treatment. As patients proceeded through the algorithm, they were asked questions dependent on their previous answer, resulting in a different number of respondents per question. Patients were taking other treatment for moderate pain (43/142, 30.3%) or for severe pain (34/74, 45.9%).

### Evaluation of the PDSS Web App

**Figure 3:**
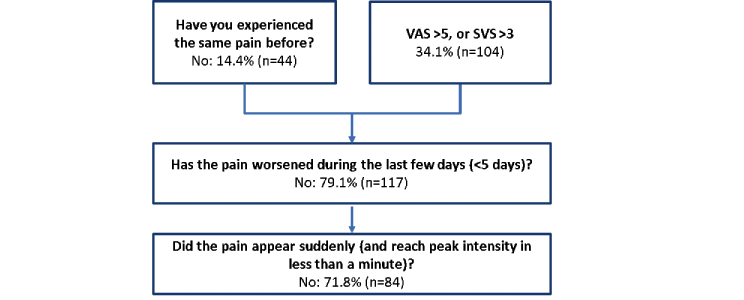
Description of patients’ pathways for the 148 patients with high pain intensity or unusual pain who did not need referral to emergency medical services. SVS: simple verbal scale; VAS: visual analog scale.

Of the 305 patients asking for immediate OTC analgesics, pain was mild in 60.3% (n=184) of patients, moderate in 24.3% (n=74) of patients, and severe in 15.7% (n=48) of patients. Some patients (n=44, 14.4%) had not previously experienced the same type of pain, and nearly half of the patients (n=148, 48.5%) described their pain as one that they had not experienced before or as one with an intensity of more than 5 on the VAS or more than 3 on the SVS (Figure 3).

Due to the algorithm, 157 patients were asked when the pain had started. The majority of patients’ pain was recent and had started earlier than 4 days prior (142/157, 90.4%). Red flags (pain with sudden onset or with increasing intensity) were identified in 64 patients, leading to specific advice and either a referral to their GP or to urgent medical services (Table 1).

At the end of the consultation, with the help of the PDSS web app, patients would either receive a referral for urgent medical services or a recommendation to make an appointment with their GP, or the pharmacy personnel member would discuss and dispense an appropriate OTC analgesic (paracetamol, ibuprofen, or aspirin). Following all 435 consultations using the PDSS web app, 79.5% (n=346) of patients only required OTC analgesics and pharmacy advice, 17.0% (n=74) were referred to urgent medical services, and 3.5% (n=15) were referred to the care of their GP (Table 2). Across all 435 consultations, paracetamol was the most common analgesic dispensed by a pharmacist (n=275, 63.2%), followed by ibuprofen (n=136, 31.3%), and aspirin (n=24, 5.5%). Among the patients who did not need a further referral, paracetamol was again the most common analgesic dispensed by a pharmacist (paracetamol: 178/346, 51.4%; ibuprofen: 139/346, 40.2%; aspirin: 28/346, 8.1%). However, among patients referred to urgent medical services, ibuprofen was more frequently prescribed (37/74, 50.0%) versus paracetamol (30/74, 40.5%) and aspirin (7/74, 9.5%).

**Table 1:** Headache pain characteristics of patients in need of immediate pain relief at the pharmacy.

		Value, n/N (%)
Patients using >2 concomitant medications		78/435 (17.9)
**Patients regularly taking analgesic medications**		109/305 (35.7)
	For moderate pain	43/142 (30.3)
	For severe pain	34/74 (45.9)
**Patient-reported assessment of pain**		
	Mild	157/261 (60.2)
	Moderate	63/261 (24.1)
	Severe	41/261 (15.7)
**Patients with experience of the same pain**		
	Yes	261/305 (85.6)
	No	44/305 (14.4)
**Patients asked if pain lasting >4 days**		
	Yes	15/157 (9.6)
	No	142/157 (90.4)
Patients with no prior experience of the same pain or pain with an intensity >5 (VAS^a^)		148/305 (48.5)
**Red flags identified**		
	Intensity of pain increased in past 5 days	31/148 (20.9)
	Sudden onset of pain	33/117 (28.2)

^a^VAS: visual analog scale.

**Table 2:** Management outcomes of all people who entered the pharmacy for headache.

Type of support given		Patients, n (%) (N=435)
**Pharmacist advice**		346 (79.5)
	Pharmacist advice + paracetamol	229 (52.6)
	Pharmacist advice + ibuprofen	97 (22.3)
	Pharmacist advice + aspirin	20 (4.6)
**Referral to urgent medical services**		74 (17.0)
	Referral to urgent medical services + paracetamol	37 (8.5)
	Referral to urgent medical services + ibuprofen	35 (8.0)
	Referral to urgent medical services + aspirin	2 (0.5)
**Referral to GP^a^**		15 (3.5)
	Referral to GP + paracetamol	9 (2.1)
	Referral to GP + ibuprofen	4 (0.9)
	Referral to GP + aspirin	2 (0.5)

^a^GP: general practitioner.

### Pharmacist and Patient Perception of the PDSS Web App

Out of all 435 patient–pharmacy personnel consultations, 119 patient questionnaires and 45 pharmacy personnel questionnaires (9 questions: n=22 pharmacies, New Aquitaine=4, Hauts de France=18; 12 questions: n=16 pharmacies, New Aquitaine=5, Hauts de France=11) were completed to assess satisfaction with the PDSS web app. The use of the PDSS web app during the consultation was very well accepted, with high levels of satisfaction reported by both pharmacy personnel and patients. All 45 pharmacy personnel were satisfied or very satisfied with the PDSS web app (Table 3). The majority considered the PDSS web app helpful to their clinical practice (34/44, 77.3%) and to the patient (38/44, 86.4%). Patient-pharmacy personnel consultation with the PDSS web app modified the management of patients’ headache by the pharmacy personnel in 64.4% of cases (29/45). Most of the 119 patients were very satisfied with the advice given by the pharmacy (n=96, 80.7%) and with the quality of information provided (n=92, 77.3%), and 100% of patients were mostly or completely reassured during the visit. Nearly all patients (n=115, 96.6%) declared they would follow the advice and recommendations given by the pharmacy (Table 4).

**Table 3:** Pharmacy personnel perception of the pharmacy decision support system web app.

Question asked		Response, n (%) (N=45)
**How satisfied were you with the digital tool? (n=37)**		
	Not satisfied	0 (0)
	Satisfied	26 (70.3)
	Very satisfied	11 (29.7)
**Do you think that use of this digital tool improved your quality of care? (n=44)**		
	Yes	41 (93.2)
	No	3 (6.8)
**Do you think the digital tool was helpful for your practice? (n=44)**		
	Very useful	6 (13.6)
	Useful	34 (77.3)
	Somewhat useful	4 (9.1)
	Not useful	0 (0)
**Do you think the digital tool was helpful for your patients? (n=44)**		
	Very useful	3 (6.8)
	Useful	38 (86.4)
	Somewhat useful	3 (6.8)
	Not useful	0 (0)
**Have you followed the recommendations of the digital tool? (n=44)**		
	Yes	43 (97.7)
	No	1 (2.3)
**Would you have managed the patient the same way had the digital tool been unavailable? (n=45)**		
	Yes	16 (35.6)
	No	29 (64.4)

**Table 4:** Patient perception of the pharmacy decision support system web app.

Response by question asked		Response, n (%) (N=119)
**Overall, how satisfied were you with the advice given by the pharmacist during your visit?**		
	Not satisfied	0 (0)
	Satisfied	27 (22.7)
	Very satisfied	92 (77.3)
**Did you feel comfortable and reassured during your visit?**		
	Not at all reassured	0 (0)
	Not really reassured	0 (0)
	Mostly reassured	87 (73.1)
	Completely reassured	32 (26.9)
**Will you follow the advice given by your pharmacist?**		
	Yes	116 (97.5)
	Partially	3 (2.5)
	No	0 (0)

## Discussion

### Principal Findings

This pilot study demonstrated that use of a PDSS web app in community pharmacies for the management of mild, moderate, and severe headache disorders is a valuable tool, both to pharmacy personnel and patients. The PDSS web app effectively identified patients to be referred to urgent medical services or to their GP for further care. Pharmacists generally reported that the PDSS web app modified their management of the patient. The PDSS web app was generally well accepted and both pharmacy personnel and patients were satisfied with the tool.

### Self-Medication With OTC Drugs

Pain is a highly prevalent symptom and is often associated with self-medication [17], with 48.2% of a community sample population using medication for pain relief [18]. According to the association of Nères (representing pharmaceutical companies which manufacture and sell self-medication products in France), sales of OTC pain medication for self-medication increased by 5.3% in 2021 versus 2020 [19]. Self-management of chronic pain conditions can provide marginal short-term and long-term benefits to the patient [20], but many patients express substantial concern over the need for pain medication and the potential for harmful effects [17]. Access to OTC medications has increased over the past decade due to switches in status from prescription only to OTC and the increased availability of OTC drugs (according to local regulations). In France in 2013, 500 million boxes of paracetamol were sold overall, and about 22,000 boxes were sold per pharmacy. Due to the risks of incorrect use and overdosing, the ANSM (Agence nationale de sécurité du médicament et des produits de santé; French National Drug and Health Products Safety Agency) requested pharmacists to have a greater role in the availability of OTC analgesics [19,21].

### The Role of Pharmacists in OTC Management

Pharmacists and “pharmacist-led medication” are crucial for the improvement of pain management and the reduction in the misuse of OTC drugs. As pharmacists have a key role in patient care being positioned directly between the patient and the physician, the public needs full confidence in the role of the pharmacist. Studies have highlighted the future potential role of pharmacist-led intervention in countries such as Canada, where a cohort study of patients with chronic pain demonstrated a low level of patient satisfaction with pain treatment in a primary care setting. This study also highlighted the potential value of pharmacists’ role in patient education, discussion of barriers and attitudes toward pain and its treatment, and monitoring pain-related disability [22]. As members of medication management teams who are easily accessible to patients, pharmacists are necessary for identifying inaccurate self-diagnoses, educating patients, and providing referral support [5,23]. This could help to ease the burden on GPs and emergency services and help the patient to select the correct treatment and right care path. The feasibility of pharmacist-led intervention for chronic pain, through assessment and adjustment of prescriptions, has been demonstrated in a previous study, with the recommendations made by the pharmacist often being implemented by the GP [24]. Furthermore, in a review of 5 randomized clinical trials, a pharmacist-led medication review reduced pain intensity, improved physical function, and improved patient satisfaction in patients with chronic pain [25].

In order to help patients, it is crucial for pharmacists to communicate effectively and gain important insight into the needs of the patient to reach positive outcomes [26,27], for example, in the dispensation of the appropriate medication or the referral to further care where appropriate. Previous evidence has suggested that a suboptimal management of OTC medications in community pharmacies is due to a lack of information gathered from the patient [28]. This study has demonstrated that the PDSS web app facilitates communication with and management of patients in the pharmacy.

Providing the patient with accurate medical advice is also key to managing pain disorders such as headache. Medication overuse is a common issue in patients with headache disorders and can cause an existing acute headache to become chronic, which is referred to as a medication-overuse headache [3]. In a study of patients with acute headache recruited at pharmacies, only 14.5% had been advised to limit their intake of treatments [29]. In the context of migraine, few self-medicating patients or patients with migraine treated by GPs are considered to be in possession of the correct treatment [30]. The Global Burden of Disease study has shown that in 2019, migraine alone was the second leading cause of disability and the leading cause of disability among women under 50 years of age [31].

Tools have previously been developed to improve screening during patient-pharmacist consultations for OTC medications, for example, a self-administered questionnaire which was shown to be a valid and reliable tool for the screening of migraine headaches in a primary care setting [32]. The use of PDSSs has been demonstrated for other conditions, for example in allergic rhinitis and conjunctivitis [8], lower back pain [9], and migraine [10]; however, to our knowledge, the PDSS web app evaluated in this study is the first to be developed for pharmacist use.

### Effect of the COVID-19 Pandemic

There was an effect of the COVID-19 pandemic on this study, with the start date in the Hauts de France region being delayed by 6 months, as pharmacies were not able to implement additional practices other than screening and vaccination for COVID-19. The study period was then extended to 4 months rather than 2 months as originally planned. In addition, a cross-sectional study of 431 people via questionnaire indicated that people became more reliant on pharmacies for medication during the COVID-19 pandemic [33].

### Strengths and Limitations

The PDSS web app developed for this study focuses on headache only, thus reducing the scope of this study in the context of self-medication. However, due to the similarities in the counseling process for all OTC indications, the results are likely similar in other areas of self-medication. Our study supports the implementation of the PDSS web app across more pharmacies in France and across the globe; however, the lack of qualitative data to show how management of patients was altered and which aspects of the PDSS web app require improvement is a limitation of this study that should be addressed in future trials. Furthermore, the anonymity of the PDSS web app does not allow a link between the patient data (eg, pain characteristics) and their satisfaction to be made. All patients preferred to fill in the paper form of the questionnaire, (ie, not the online version of the form), leading to possible bias regarding the homogeneity of patient type. Likewise, the uptake of the PDSS webapp will be voluntary for pharmacies, leading to possible bias toward pharmacies and personnel that are willing to adopt new technology as well as pharmacists specializing in pain management.

### Future Directions

Self-medication is an important part of a patient’s treatment plan and should be encouraged, as it gives patients control over their treatment. Recommendations from health care professionals, particularly pharmacists, therefore need to be carefully individualized to the patient to ensure safety. With this in mind, studies should be conducted to identify patient profiles, behaviors, and characteristics associated with responsible self-medication and, consequently, to develop multichannel digital tools to allow pharmacists and GPs to quickly assess the level of risk associated with patient self-medication. Computerized tools such as the PDSS evaluated in this study do not aim to replace health care professionals in decision-making during patient management but rather aim to meet the needs of the patient regarding safe self-medication. As such, future studies could benefit training programs for pharmacists and pharmacist-led medication and better emphasize and inform the “red flags” that indicate a patient is at risk of drug misuse or misdiagnosis. Such “red flags” should be screened for by the PDSS web app so that patients are diverted to appropriate further care, such as their general practitioner or urgent medical services. Larger studies should measure the impact of the PDSS web app on the lives of the patients and the safety of the pharmacist recommendations. Interventional trials should also examine the impact of the mandatory training prior to PDSS web app use on pharmacist satisfaction; the time dedicated to training or certain aspects of the training given (ie, the scientific background and “red flags” that pharmacists may already be aware of) may decrease the satisfaction with the PDSS web app. However, the algorithm itself might have helped to teach pharmacists and pharmacy personnel how to manage headache in real-world situations and more easily and efficiently during an epidemic such as that of COVID-19. This may in turn increase satisfaction with the PDSS web app although this should be examined in future trials. The COVID-19 epidemic has shown the crucial positioning of the pharmacist when a patient first seeks medical help. This type of PDSS web app can help pharmacy teams better take care of patients and manage them, particularly as algorithms are further digitalized.

### Conclusions

Community pharmacies are valuable in the study and management of headaches. Proper patient-pharmacist counseling is crucial to inform next steps of treatment for headache. A newly developed PDSS web app effectively guided pharmacy personnel recommendations for self-medication with analgesics and identified patients who required referrals to specialist care. The PDSS web app was well accepted by patients and pharmacy personnel, and further studies are warranted to optimize such tools for OTC self-medication.

## Appendix

Multimedia Appendix 1 Recommended management algorithm for use by pharmacy for patients with headache (initial consultation and for nonsevere pain).

Multimedia Appendix 2 Recommended management algorithm for use by pharmacy for patients with severe headache or red flags.

## Abbreviations

ANSM: Agence nationale de sécurité du médicament et des produits de santé
ARS: Agences Régionales de Santé
GAMP: Good Automated Manufacturing Practice
GDPR: EU General Data Protection Regulation
GP: general practitioner
OTC: over the counter
PDSS: pharmacy decision support system
SFETD: French Scientific Society for Pain
SFSPO: Société Francophone des Sciences Pharmaceutiques Officinales
SVS: simple verbal scale
URPS: Regional Unions of Health Professionals
VAS: visual analog scale

## Data Availability

The data sets generated during or analyzed during the current study are available from the corresponding author upon reasonable request.
