# Supplementary material for: A Computerized Pharmacy Decision Support System (PDSS) for Headache Management: Observational Pilot Study
Source: Interact J Med Res. 2022 Nov 25;11(2):e35880. doi: 10.2196/35880 (PMC9736760; doi:10.2196/35880)
Supplement: Multimedia Appendix 1 [file ijmr_v11i2e35880_app1.docx]

# Multimedia Appendix 1. Recommended management algorithm for use by pharmacy for patients with headache (initial consultation and for non-severe pain)


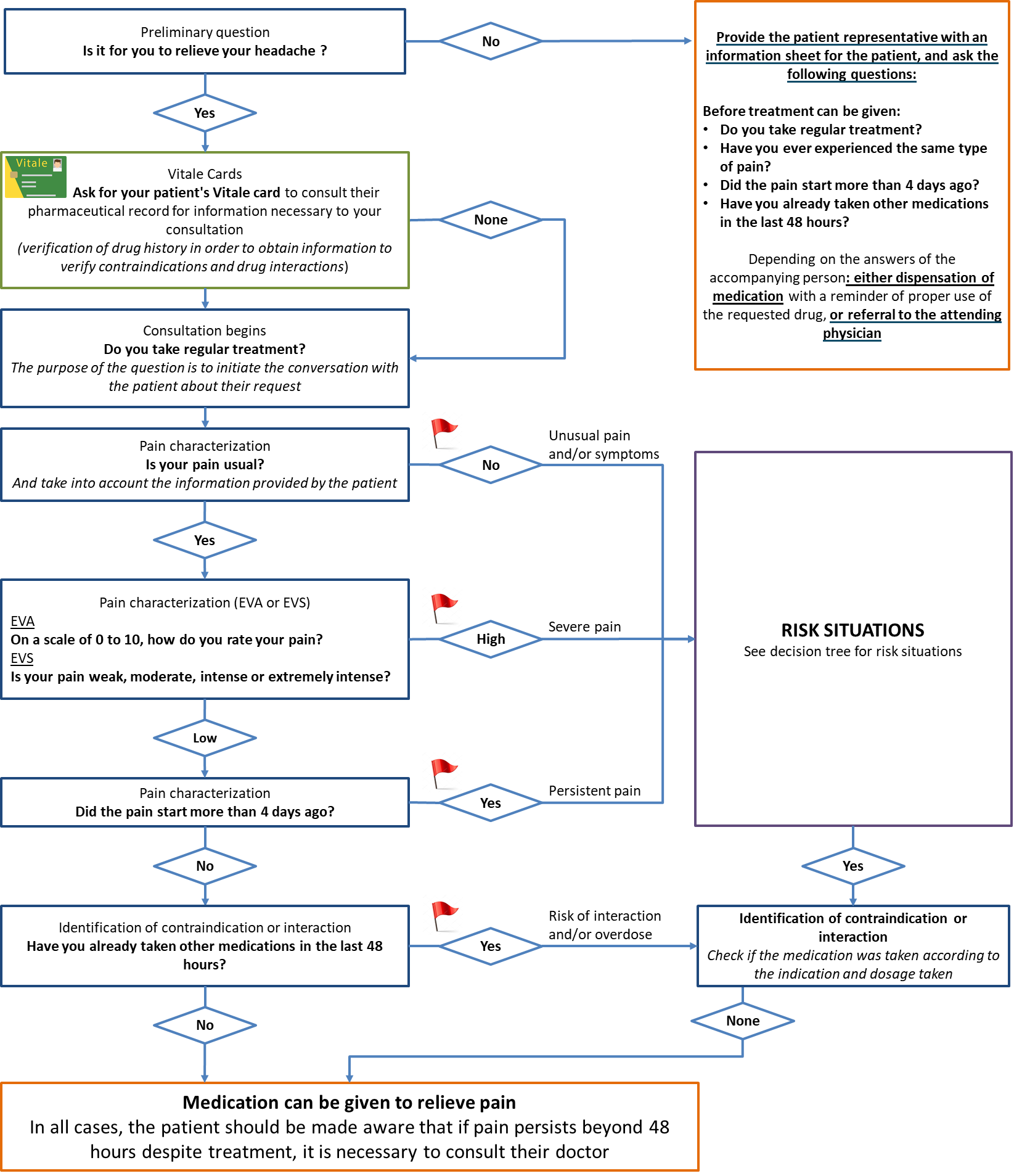


EVA, visual analogue scale; EVS, simple verbal scale

A high EVA or EVS score is considered to be 5–10 (EVA) or <3 (EVS)
