# Supplementary material for: A Computerized Pharmacy Decision Support System (PDSS) for Headache Management: Observational Pilot Study
Source: Interact J Med Res. 2022 Nov 25;11(2):e35880. doi: 10.2196/35880 (PMC9736760; doi:10.2196/35880)

Multimedia Appendix 2. Recommended management algorithm for use by pharmacy for patients with severe headache or red flags


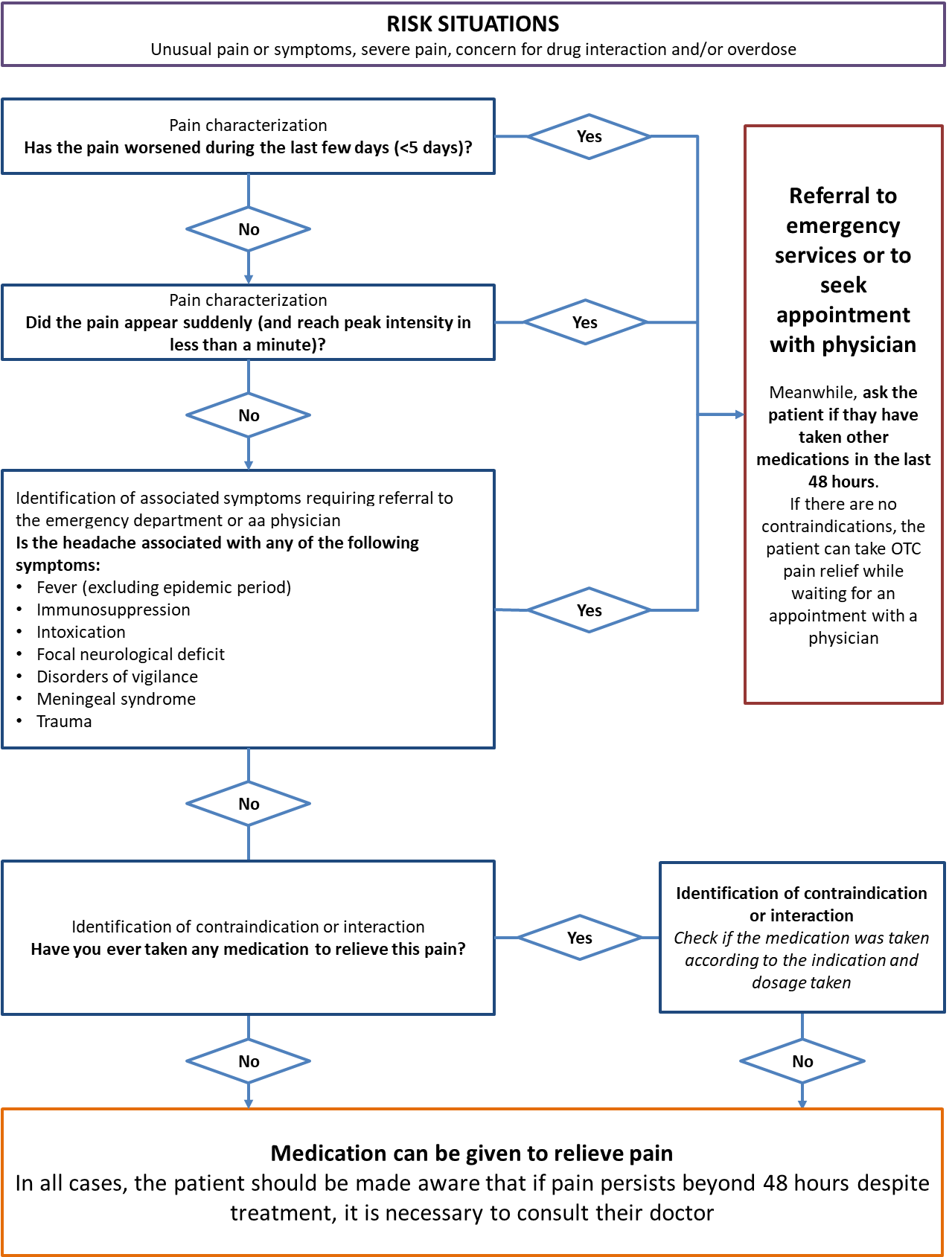

Supplement: Multimedia Appendix 2 [file ijmr_v11i2e35880_app2.docx]
